# Histocompatibility and Long-Term Results of the Follicular Unit-Like Wigs after Xenogeneic Hair Transplantation: An Experimental Study in Rabbits

**DOI:** 10.5402/2011/134502

**Published:** 2011-04-06

**Authors:** Yu Sun, Feng Lu, Ge Liu, Zhi-Dan Zhang, Zijie Zhang, Zhi-Qi Hu

**Affiliations:** Department of Plastic Surgery, Nanfang Hospital, Southern Medical University, Guangzhou, Guangdong Province, 510515, China

## Abstract

*Objective*. This study was designed to observe the histocompatibility and long-term results of wigs after xenogeneic hair transplantation and to explore the possibility of industrial products in clinical application. *Methods*. The human hair and melted medical polypropylene were preceded into the follicular unit-like wigs according to the natural follicular unit by extrusion molding. 12 New Zealand rabbits were used as experimental animals for wigs transplantation. The histocompatibility of polypropylene and human hair was observed by H&E staining and scanning electron microscope. The loss rate of wigs was calculated to evaluate the long-term result after transplantation. *Results*. Mild infiltration by inflammatory cells around the polypropylene and human hair were seen during the early period after transplantation, accompanied with local epithelial cell proliferation. The inflammatory cells were decreased after 30 days with increased collagen fibers around the polypropylene and human hair. The follicular unit-like wigs maintained a good histocompatibility in one year. The degradation of hair was not significant. The loss rate of wigs was 4.1 ± 4.0% in one year. The appearance of hair was satisfactory. *Conclusions*. We successfully developed a follicular unit-like wigs, which were made of xenogeneic human hair with medical polypropylene, showing a good histocompatibility, a low loss rate, and satisfactory appearance in a year after transplantation. The follicular unit-like wigs may have prospective industrial products in clinical application.

## 1. Introduction

 The follicular unit transplantation is the most effective technique of treating baldness and widely used in clinical practice at present. This technique redistributes the follicular units in the balding area and can obtain good long-term stability with a natural appearance. However, this technology is severely restricted in patients with large alopecia because of limited self-donation.

 Since 2006, our research group had carried out a series of studies. And we designed a type of portable wigs [1, 2] (patent number 200620057171). The portable wigs were expected to solve the serious shortage of patient's self-donation for the clinical needs. However, in the previous preliminary study, the products were found to have some limitations in clinical application and require further improvement.The current study was aimed at to improve our previous designation and try to develop a follicular unit-like wigs with human hair and also to investigate the histocompatibility, long-term results of wigs after transplantation. Our final goal was to explore the possibility of industrial products in clinical application.

## 2. Material and Methods

### 2.1. Material and Equipments

Medical polypropylene (Taiwan Chemical Fiber Co., Ltd., Model: K4515), xenogeneic hair from adult female of 22–30 year old, electric heater, industrial thermometer, pressure steam sterilizer, stainless steel handle, optical microscope, and biological microscope.

### 2.2. Designation of the Follicular Unit-Like Wigs

The base of graft was square-shaped with a visible barb. The angle between the barb and the base was 20~40 degree. Human hair was closely surrounded by the polypropylene base. Each graft contains 1~4 hairs. The length of hair can be customized according to patient's need. The length of hair used in current experiments is about 14 cm.

### 2.3. Manufacture of Follicular Unit-Like Wigs

 Preparation of hair: hair from healthy females was soaked in 75% alcohol for 2 hours, and rinsed with 0.9% NaCl, then immerged in 2% glutaraldehyde for 20 minutes, and rinsed many times in 0.9% NaCl to wash off any residual glutaraldehyde in human hair.

**Figure 1:**
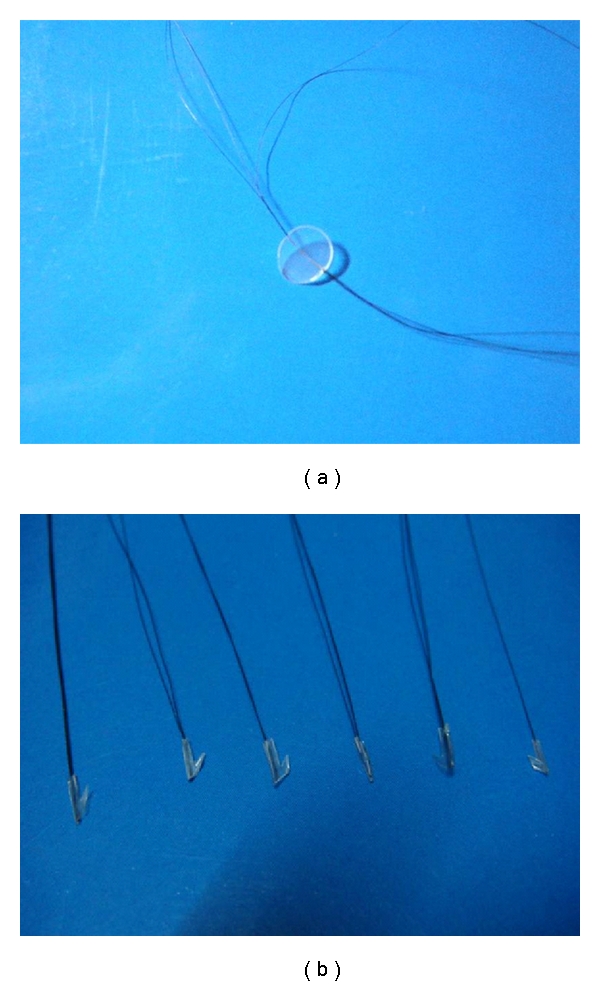
(a) An intermediate product of graft with hair. (b) Six finished products (the follicular-unit like wigs) with a fish-hook-like base.

 The followings are steps to prepare the follicular unit-like wigs (1) Combination of hair and polypropylene performed under a sterile bench: first, an electric furnace was heated to a suitable temperature (160 ± 10°C), and the surface temperature was probed by an industrial thermograph. The polypropylene particles were placed on the stainless steel and heated on the furnace surface until it became melt state. The polypropylene was removed out when it became in melted state; the melt-state polypropylene is very soft, so the hair was easily introduced into the melt-state polypropylene by gently pressing both sides of hair. Another stainless steel was used to press the melt polypropylene and hair; the hair was tightly enclosed by the melt polypropylene after pressing of stainless steel and finally demounted at room temperature. The obtained intermediate products were lamellar (showed in Figure 1(a)). (2) Elaboration of intermediate products: one side of the polypropylene was cut off with size 11 blade along the longitudinal axis of the hair and the opposite side was cut off partly. Therefore, one barb was elaborated at the base of the stump, and the angle of the barb was about 30 degree with a fish-hook shape. The length of the stump was about 4 mm, the width was about 1 mm, and the thickness was 300~400 *μ*m. One to four hairs were encapsulated in the base (showed in the Figure 1(b)).

### 2.4. Experimental Animals

 12 New Zealand rabbits (from Experimental Animal Center of Southern Medical University) weighing 2,500 to 3,400 g were used.

### 2.5. Wigs Transplantation

 The rabbits were anaesthetized with 3.0% sodium pentobarbital (30 mg/kg) and immobilized on an operating table with adhesive tape; the scalp fur was shaved off with razor blade, and the scalp skin was disinfected with 0.3% povidone-iodine and 75% alcohol. Punch needle for hair transplantation was used to create slits (6~8 mm in depth) in the scalp. a fine ophthalmic forceps were used to implant the wigs into the slits. The length of hair embedded in the skin was about 2~3 mm. Total 40 unit wigs were implanted into each rabbit. 10 wigs were with 1 hair each, 10 wigs with 2 hairs each, 10 wigs with 3 hairs each, and 10 wigs with 4 hairs each. After the wigs implantation, the rabbits were randomly divided into A and B groups (*n* = 6). Group A was used for long-term outcomes after transplantation and Group B for observation of histocompatibility. Head washing was given a week post operation.

### 2.6. Evaluation of Physical Strength of Wigs

Scanning electron microscopy was used to observe the contact surface between polypropylene and hair, and anti tensile force assay was used to test the stability of the wigs.

### 2.7. Evaluation of Long-Term Outcomes of Transplantation

The wound healing of these slits postoperative, was evaluated for swelling, ulceration, and infection. The appearance of the wigs was also evaluated. The loss rate of wigs was calculated at 1 week, 1 month, 3 months, 8 months, and 12 months following transplantation in group A. The total loss rate was calculated at 12 months. 

### 2.8. Histological Evaluation

Biopsies in these rabbits of group B were performed at 1 week, 1 month, 3 months, 8 months, and 12 months after implantation under sterile conditions. The specimens were fixed in 10% formalin, paraffin-embedded and sectioned according to established protocol in our lab. The histological changes around the wigs in those specimens were studied by H&E staining. Some specimens were fixed in 2.5% glutaraldehyde, dried at critical point, and observed by scanning electron microscope.

## 3. Results

### 3.1. Evaluation of Physical Strength of the Wigs

**Figure 2:**
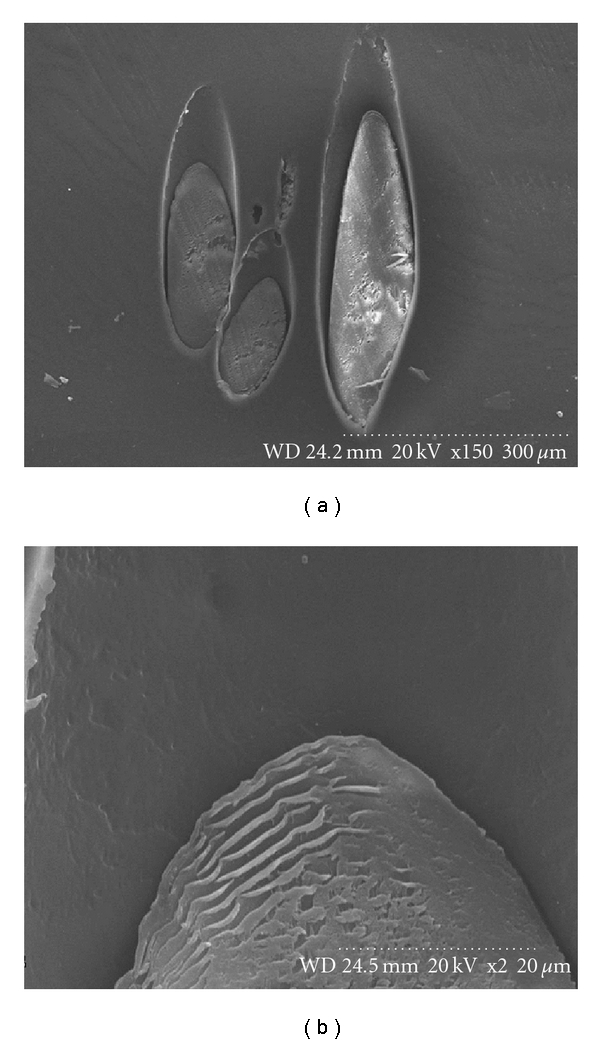
Observation of graft by scanning electron microscope. (a) Human hair was enclosed tightly by polypropylene, and no significant space was seen in the contact surface (×150). (b) The human hair cuticle impress traces were easily identified on the wall of polypropylene. The structure of human hair was clear and the cuticle of hair imbricate superimposed closely (×2000).

The scanning electron microscope showed that the human hair was enclosed tightly by polypropylene. No significant space was seen in the contact surface (Figure 2(a)). The human hair cuticle impress traces were easily recognized on the wall of polypropylene. The structure of human hair was clear and the cuticle of hair imbricate superimposed closely as shown in Figure 2(b). Anti-tensile force assay showed that the binding of polypropylene with hair was very tight. Even if hair was pulled off, it still cannot be easily pulled out from the polypropylene base.

### 3.2. Long-Term Results of Transplantation

**Figure 3:**
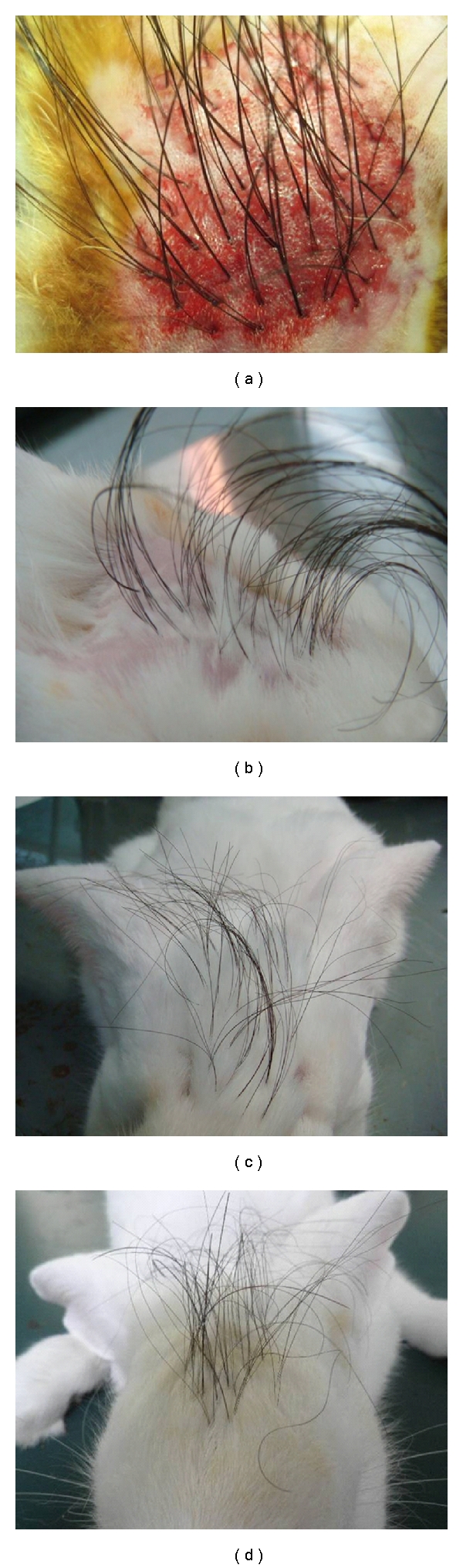
The appearance of wigs after implantation. (a) Immediate implantation. (b) 1 month after implantation. (c) 8 months after implantation. (d) 12 months after implantation.

 The follicular unit-like wigs were small and could be implanted as follicular unit transplantation. The follicular unit-like wigs showed satisfactory appearance with natural orientation and even distribution after transplantation. The slits created from implantation were slightly red and swollen in the early postoperative period. However, these changes disappeared after one week. No hematoma, infection, exudation, ulcer, and necrosis were observed within 12 months (Figure 3).

 The total loss rate of follicular unit wigs in 6 rabbits after implantation was 4.1 ± 4.0% in 12 months. Their average loss rates were 2.5% at 1 week, 1.25% between 1 week and 1 month, and 0.4% between 1 month and 3 months after implantation. The standard deviations of loss rates were 2.7%, 2.0%, and 0.1%, respectively. The loss rate was 0 between 3 to 12 months, probably due to the healing of slits and the role of polypropylene barb which were anchored firmly in the subcutaneous tissue. Loss rates have been shown in Table 1.

### 3.3. Histological Evaluation

**Figure 4:**
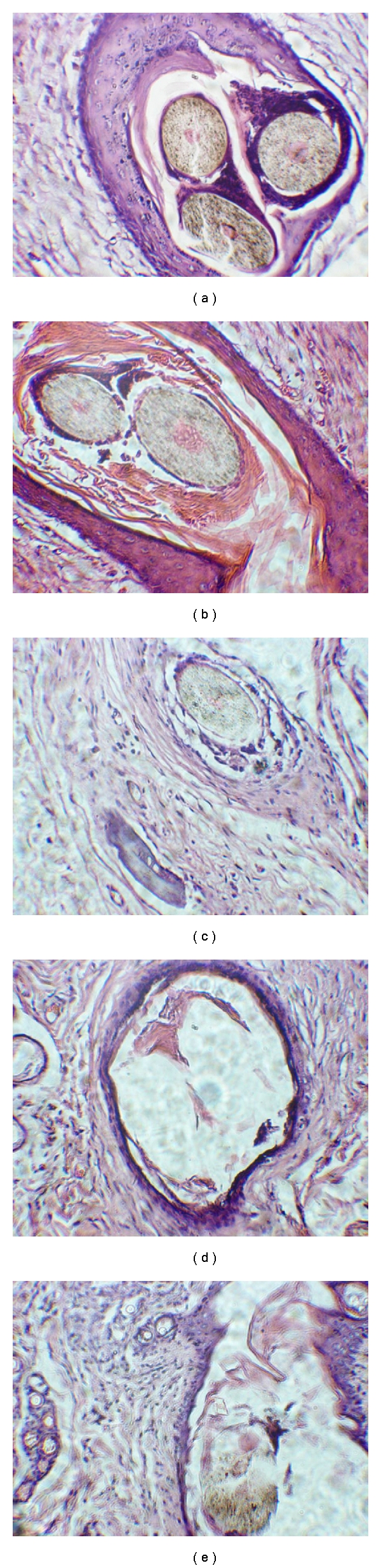
The histological observation of hair (H&E stain, 10 × 10). (a) 1 week after implantation, there was a mild infiltration with a small number of neutrophils and lymphocytes. The multinucleated giant cells were present around polypropylene and the hair. The epithelial cells were observed around the hair. (b) At 1 month, the neutrophils, lymphocytes, and multinucleated giant cells were reduced, while fibroblasts were increased. More epithelial cells surrounded the hair. (c, d, e) At 3, 8 and 12 months after implantationno obvious infiltration of inflammatory cells was identified and epithelial cells were still seen. Many fibroblasts and collagen fibers were seen around the hair and polypropylene. Infiltration by lymphocytes and reject reaction were not evident at 12 months.

**Figure 5:**
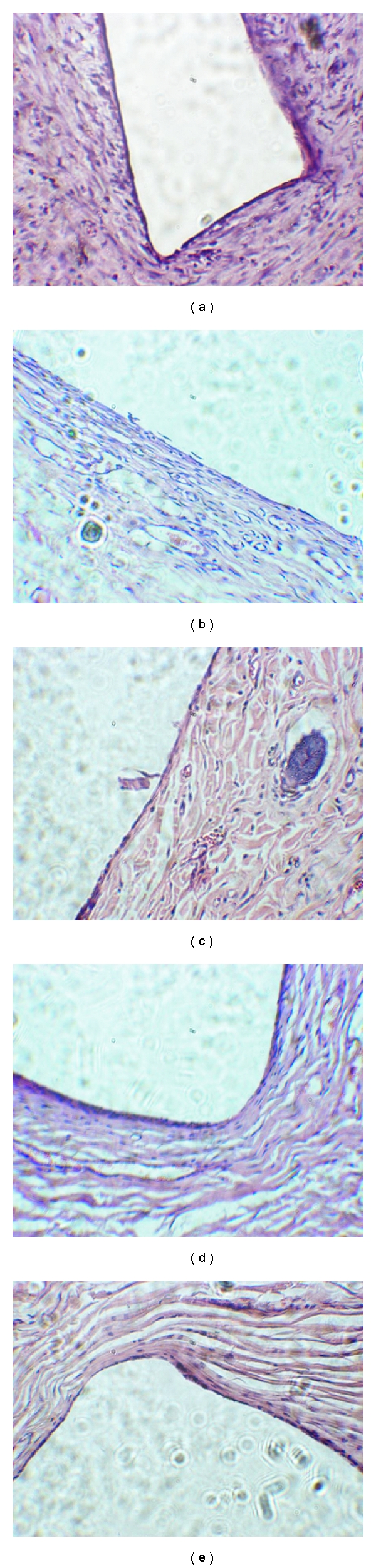
The histological observation of polypropylene (H&E stain, 10 × 10). (a) 1 week after implantation. The white area represented polypropylene. There was infiltration of a small number of neutrophils and lymphocytes. The multinucleated giant cells were observed around the polypropylene, and the epithelial cells were not observed. (b) At 1 month, the neutrophils, lymphocytes, and multinucleated giant cells were decreased, while fibroblasts were increased and de novo blood vessels were observed. (c, d, e) At 3, 8, and 12 months after implantation, there was no obvious inflammatory infiltration.

H&E staining histological evaluation: there were mild inflammatory infiltration by neutrophils and lymphocytes and small vessel congestion in 1 week after implantation. Multinucleated giant cells were observed around the polypropylene and hair. Slight epithelial cell proliferation was observed around the hair. Neutrophils, lymphocytes, and multinucleated giant cells were reduced in 1 month while fibroblasts increased and more epithelial cells surrounded the hair. No significant histological changes were evident in 3 to 8 months following implantation. In comparison with the changes at first week, the infiltration by inflammatory cells almost disappeared. The formation of fibroblasts and collagen fibers surrounded the hair and polypropylene had strengthened. Much more epithelial cells were present around the hair. At 12 months, many fibroblasts and collagen fibers were seen around the hair and polypropylene. The inflammatory infiltration by lymphocytes and reject reaction were not identified (Figures 4 and 5).

**Figure 6:**
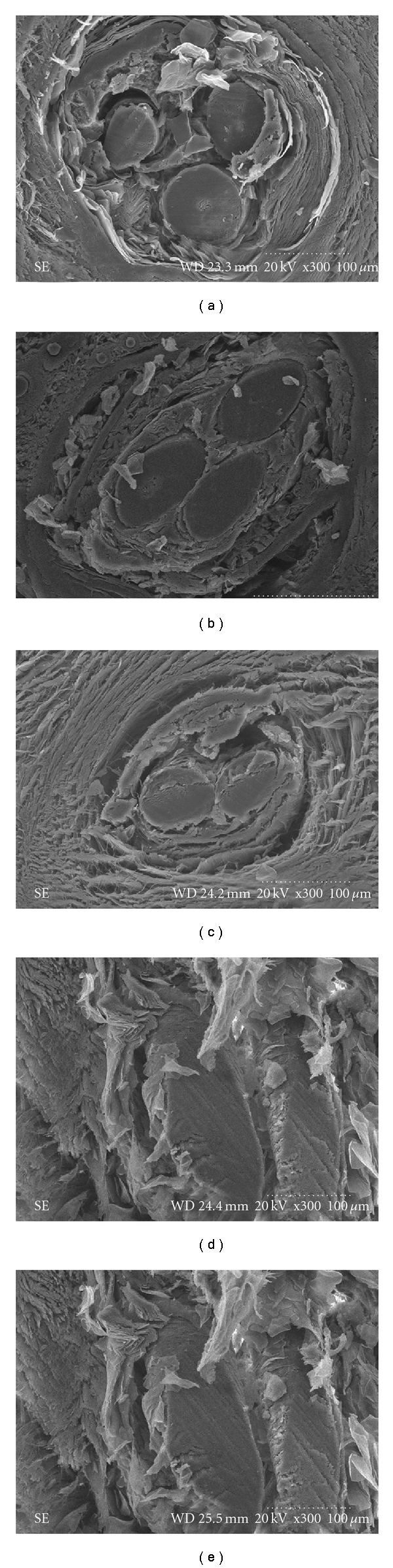
Observation of hair with scanning electron microscope (×300). (a) At 1 week after implantation, a certain number of epithelial cells and fibroblasts surrounded the hair. (b) At 1 month, epithelial cell and fibroblasts still existed. (c, d, e) After 3, 8, and 12 months implantation, the fibroblasts were increased, but the epithelial cells were somewhat reduced. At 12 months, the hair structure was still clear, and no degradation was identified.

**Figure 7:**
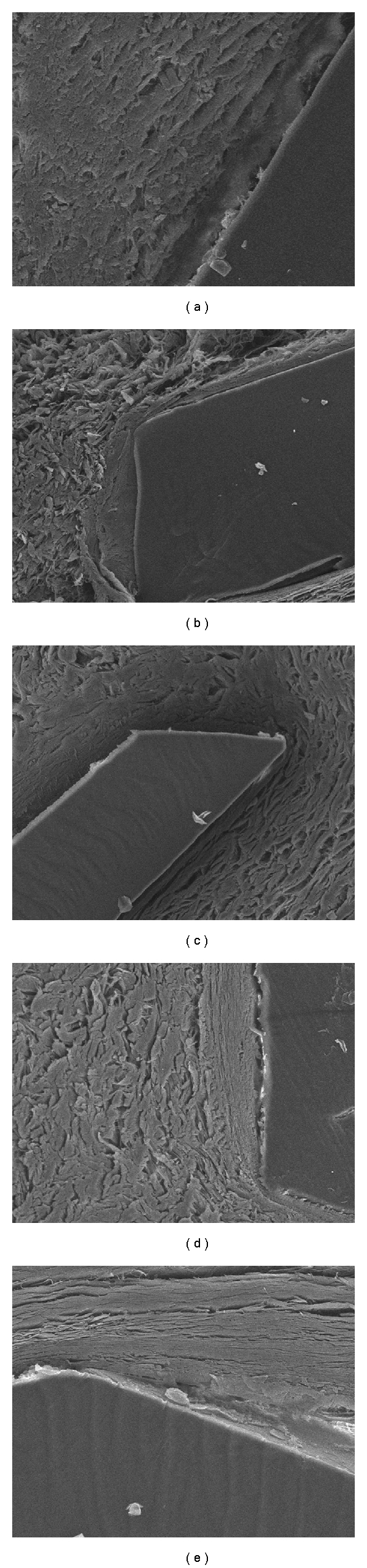
Polypropylene under scanning electron microscope (×300). (a) 1 week after implantation, a certain number of fibroblasts surrounded the polypropylene, but the epithelial cells were not observed. (b) 1 month after implantation, the fibroblasts were increased obviously, and the contact between polypropylene and tissue was firm. (c, d, e) 3, 8, and 12 months after implantation, the fibroblasts were increased, and no tissue rejection and necrosis were present.

 The scanning electron microscope histological evaluation demonstrated a homogeneous, smooth, and translucent polypropylene closely integrated with the surrounding tissue. No tissue dissolution and rejection were found. At 1 week the hair was surrounded by epithelial cells tightly. Fibroblast hyperplasia around the polypropylene was not seen definitively. However, fibroblast hyperplasia around the polypropylene and hair was more dynamic at 1 month while epithelial cells around the hair were reduced slightly. At 6 to 12 month, significant fibroblast hyperplasia and collagen fibers were seen. A good histocompatibility was maintained with no evidence of tissue necrosis and rejection. Hair degradation was not observed at the end of 12 month. The structure of hair was still clear (Figures 6 and 7).

## 4. Discussion

 Currently, the most effective technique of treatment alopecia is follicular unit transplantation. This technique, however, is not satisfying to patients with large alopecia with limited self hair donation. The only way to cover large baldness area is wearing wigs. However, the traditional wigs are not perfect. First, they are not durable and easily pulled off by extra force. Second, they are hermetic and sweltering. Third, the wigs are easily to become chaos, not convenient for combing, and not natural in appearance. Therefore, designing follicular unit-like wigs may solve the problem for patients with large baldness area. 

 Agrawal and Santiago et al. reported that Biofiber could be used to restore the scalp alopecia [3, 4]. Biofiber was a kind of artificial hair made of polyamide and was manufactured in Italy. The studies showed that it had good histocompatibility but high loss rate (18~23% per year). The mean density was only 26.7 fiber/cm^2^. It still cannot meet the needs of patients with large alopecia. 

 In 1984, Headington [5] first described the follicular unit as an anatomic entity. The follicular unit is composed of one to four terminal hairs, associated sebaceous glands, erector pili muscle, and surrounding connective tissue. This theory significantly promoted the clinical application of follicular unit dissection and hair transplantation. In our studies, this concept was adapted to design the wigs (follicular unit-like wigs). The follicular unit-like wig is similar to natural hair follicles in structure. Therefore, it is suitable for industrial production and clinical application. In our studies, polypropylene and hair were proceeded according to the follicular unit by extrusion molding and further elaborated into the follicular unit-like wigs. The mild inflammatory infiltration was found in the early period after implantation, which may be due to the surgical trauma. As the slits heal, these inflammatory cells were gradually reduced, and epithelial cells from epidermal proliferation surrounded the hair tightly and closed the slits. This phenomenon was also identified in the later period. Thereafter, it may serve as a barrier of entry for the pathogens from skin surface to deeper scalp tissue and makes the implant not infection prone. The fibrous tissue was increased obviously at 1 month, similar to other reports [6, 7]. This transition to fibrous connective tissue may be associated with the stimulation by the hair and polypropylene as a result of foreign body reaction [6, 7]. In comparison with 1 week, fibrous tissue was more dynamic, especially after 3 months. The findings from histological examination of the connective tissue around the hair and polypropylene were not decreased at 12 months suggesting that this fibrosis may be permanent and the wigs should not so easily fall out.

 At the end of 12 month, no inflammatory reaction was found. No hematoma, infection, exudation, ulcer, and necrosis were observed. The rabbit's fur around the wigs grew normally, even faster than other regions, which indicates that xenogeneic human hair is nonantigenic and does not cause immune rejection in the rabbits. The histocompatibility is acceptable. All the wigs still remained in the scalp of the rabbits in one year. The hair was not easy to break, and no significant degradation and erosion were observed by the scanning electron microscopy, likely due to special physical and chemical structure of the hair [7]. 

 The structure of follicular unit-like wigs was similar to the natural anatomy of human hair follicles. The follicular unit-like wigs were loose with an almost natural appearance and easy to clean. Moreover, the stability was high, and the loss rate decreased gradually after implantation. The total loss rate was 4.1 ± 4.0%. Compared with Biofiber, it was more permanent. As experimental animals, rabbits' skin was thinner than human scalp; therefore, high density of implantation was not attempted. The appearance of follicular unit-like wigs may be more close to nature with dense implantation in alopecia areas. 

 The final objective of this study is to develop a kind of portable wigs for the patients with limited self-donation but with normal skin alopecia area. If the androgenetic alopecia patients have enough self-donation, we think that the transplantation of autogenous hair follicular unit is a better choice. While the androgenetic alopecia patients do not have enough self-donation, they could choose to let their hair grow long enough, then reconstruct the alopecia by developing this kind of follicular unit wig which is made of autologous hair or allogeneic hair. At present, we are not sure whether or not it is useful to repair scarring alopecia owing to that the animal model in this experiment is a normal scalp instead of scar tissue. The further study is needed to confirm it.

 In summary, we successfully developed follicular unit-like wigs with human hair and polypropylene, which have a good histocompatibility, a low loss rate, and more natural appearance after transplantation. The follicular unit-like wigs may have prospective industrial products in future clinical application. It is likely to provide a new resource of hair for patients with large baldness. These patients could choose certain length of autogeneic or xenogeneic hair to customize the wigs to repair baldness instead of extraction of hair follicle by traditional technique. Therefore, the follicular unit-like wigs are more convenient for clinic practice.

## Figures and Tables

**Table 1:** The loss rate of wigs in different period.

Period	1 week	1 week~1 months	1–3 months	3–8 months	8–12 months	12 months
Loss rate	(2.5 ± 2.7)%	(1.25 ± 2.0)%	(0.4 ± 0.1)%	0%	0%	(4.1 ± 4.0)%
